# Crystal structure and Hirshfeld surface analysis of (*E*)-1-(2,6-di­chloro­phen­yl)-2-(2-nitro­benzyl­idene)hydrazine

**DOI:** 10.1107/S2056989020008567

**Published:** 2020-07-03

**Authors:** Sevim Türktekin Çelikesir, Mehmet Akkurt, Namiq Q. Shikhaliyev, Gulnar T. Suleymanova, Gulnare V. Babayeva, Nurana V. Gurbanova, Gunay Z. Mammadova, Ajaya Bhattarai

**Affiliations:** a Department of Physics, Faculty of Sciences, Erciyes University, 38039 Kayseri, Turkey; b Organic Chemistry Department, Baku State University, Z. Khalilov str. 23, AZ, 1148 Baku, Azerbaijan; c Department of Chemistry, M.M.A.M.C (Tribhuvan University), Biratnagar, Nepal

**Keywords:** crystal structure, face-to-face π–π stacking inter­actions, 2,6-di­chloro­phenyl ring, nitro-substituted benzene ring, Hirshfeld surface analysis

## Abstract

In the title compound, C_13_H_9_Cl_2_N_3_O_2_, the 2,6-di­chloro­phenyl ring and the nitro-substituted benzene ring form a dihedral angle of 21.16 (14)°. In the crystal, face-to-face π–π stacking inter­actions occur along the *a-*axis direction between the centroids of the 2,6-di­chloro­phenyl ring and the nitro-substituted benzene ring. Furthermore, these mol­ecules show intra­molecular N—H⋯Cl and C—H⋯O contacts and are linked by inter­molecular N—H⋯O and C—H⋯Cl hydrogen bonds, forming pairs of hydrogen-bonded mol­ecular layers parallel to (20). The Hirshfeld surface analysis of the crystal structure indicates that the most important contributions to the crystal packing are from H⋯H (23.0%), O⋯H/H⋯O (20.1%), Cl⋯H/H⋯Cl (19.0%), C⋯C (11.2%) and H⋯C/C⋯H (8.0%) inter­actions.

## Abstract 2

In the crystal, face-to-face π-π stacking inter­actions occur along the *a-*axis direction between the centroids of the 2,6-di­chloro­phenyl ring and the nitro-substituted benzene ring. In addition, these mol­ecules show intra­molecular N—H⋯Cl and C—H⋯O contacts and are linked by inter­molecular N—H⋯O and C—H⋯Cl hydrogen bonds, forming pairs of hydrogen-bonded mol­ecular layers parallel to (20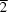).

## Chemical context   

Aryl­hydrazones and their complexes have attracted much attention because of their high synthetic potential for organic and inorganic chemistry and diverse useful properties (Maharramov *et al.*, 2009 ▸, 2010 ▸, 2018 ▸; Mahmudov *et al.*, 2010 ▸, 2011 ▸, 2014*a* ▸). The analytical and catalytic properties of this class of compounds are strongly dependent on the attached groups to the hydrazone moiety (Mahmudov *et al.*, 2013 ▸; Shixaliyev *et al.*, 2018 ▸, 2019 ▸). On the other hand, inter­molecular inter­actions organize the mol­ecular architectures, which play a critical role in synthesis, catalysis, micellization, *etc*. (Akbari Afkhami *et al.*, 2017 ▸; Gurbanov *et al.*, 2017 ▸, 2018 ▸; Kopylovich *et al.*, 2011*a* ▸,*b* ▸; Ma *et al.*, 2017*a* ▸,*b* ▸; Mahmoudi *et al.*, 2016 ▸, 2017*a* ▸,*b* ▸,*c* ▸, 2018*a* ▸,*b* ▸). New types of non-covalent bonds such as halogen, chalcogen, pnictogen and tetrel bonds or their cooperation with hydrogen bonds are able to contribute to the synthesis and catalysis, giving materials with improved properties (Mahmudov *et al.*, 2013 ▸, 2014*b* ▸, 2015 ▸, 2017*a* ▸,*b* ▸, 2019 ▸; Mizar *et al.*, 2012 ▸; Shixaliyev *et al.*, 2013 ▸, 2014 ▸). For that, the main skeleton of the hydrazone ligand should be decorated by non-covalent bond donor centre(s). In a continuation of our work in this regard, we have functionalized a new azo dye, (*E*)-1-(2,6-di­chloro­phen­yl)-2-(2-nitro­benzyl­idene)hydrazine, which provides inter­molecular non-covalent inter­actions.
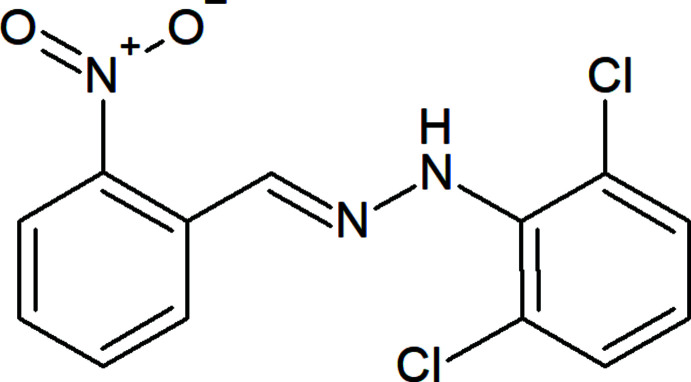


## Structural commentary   

**Figure 1:**
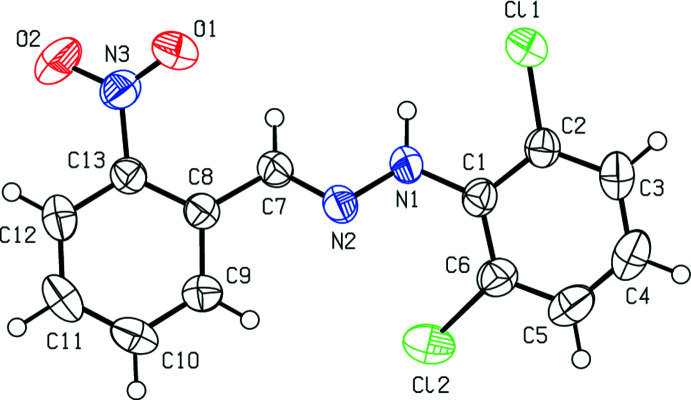
The mol­ecular structure of the title compound, showing the atom labelling and displacement ellipsoids drawn at the 50% probability level.

The title mol­ecule (Fig. 1 ▸) has an *E* configuration about the C=N bond. The 2,6-di­chloro­phenyl ring and the nitro-substituted benzene ring of the title compound are inclined at 21.16 (14)°, while the nitro group is skewed out of the attached benzene ring plane by 27.06 (18)°. The Cl1—C2—C1—N1, Cl2—C6—C1—N1, C2—C1—N1—N2, C1—N1—N2—C7, N1—N2—C7—C8, N2—C7—C8—C13, C7—C8—C13—N3, C8—C13—N3—O1 and C8—C13—N3—O2 torsion angles are 0.1 (3), 4.7 (4), −145.8 (2), 176.7 (2), 175.4 (2), 164.3 (3), −7.7 (4), −26.9 (4) and 155.7 (3)°, respectively. Two intra­molecular N—H⋯Cl and C—H⋯O contacts are present (Table 1 ▸).

## Supra­molecular features and Hirshfeld surface analysis   

**Figure 2:**
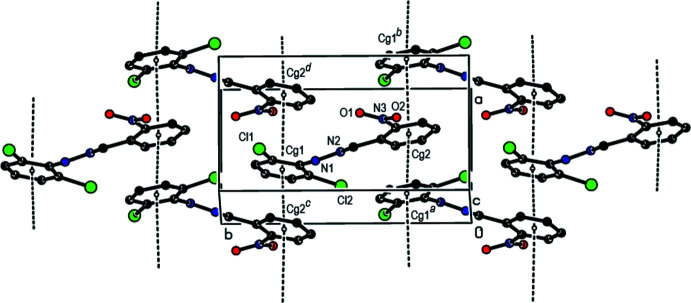
A view of π–π stacking inter­actions of in the crystal packing of the title compound. *Cg*1 and *Cg*2 are the centroids of the C1–C6 and C8–C13 benzene rings, respectively. [Symmetry codes: (*a*) 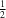 − *x*, −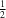 + *y*, 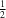 − *z*; (*b*) 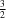 − *x*, −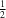 + *y*, 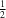 − *z*; (*c*) 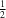 − *x*, 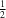 + *y*, 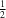 − *z*; (*d*) 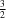 − *x*, 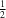 + *y*, 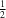 − *z*].

**Figure 4:**
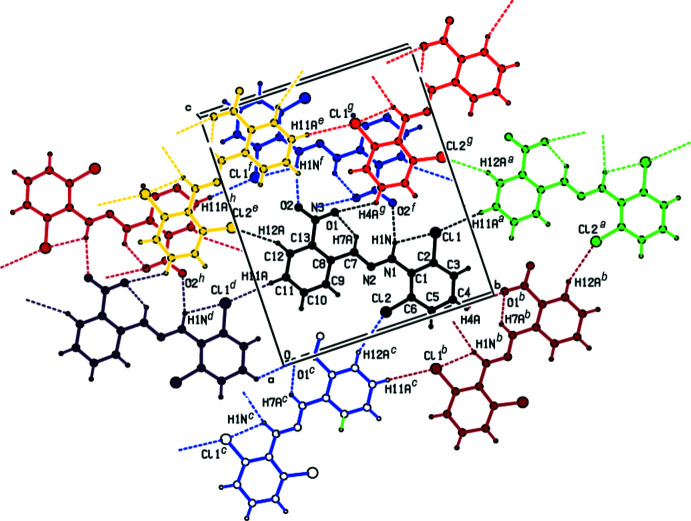
A general view of the crystal packing along the *a* axis of the title compound. Dashed lines indicate the intra­molecular N—H⋯Cl, C—H⋯O, inter­molecular N—H⋯O, C—H⋯Cl inter­actions and Cl⋯H, O⋯H contacts. [Symmetry codes: (*a*) *x*, 1 + *y*, *z*; (*b*) −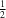 + *x*, 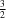 − *y*, −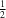 + *z*; (*c*) −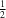 + *x*, 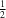 − *y*, −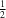 + *z*; (*d*) *x*, −1 + *y*, *z*; (*e*) 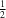 + *x*, 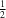 − *y*, 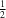 + *z*; (*f*) 1 − *x*, 1 − *y*, 1 − *z*; (*g*) 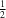 + *x*, 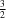 − *y*, 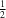 + *z*; (*h*) 1 − *x*, −*y*, 1 − *z*].

In the crystal, face-to-face π–π stacking inter­actions [*Cg*1⋯*Cg*2(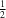 − *x*, 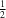 + *y*, 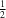 − *z*) = 3.7605 (17) Å with slippage of 1.352 Å, *Cg*1⋯*Cg*2(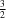 − *x*, 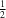 + *y*, 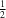 − *z*) = 3.8010 (17) Å with slippage of 1.457 Å, where *Cg*1 and *Cg*2 are the centroids of the C1–C6 and C8–C13 rings, respectively] occur between the centroids of the 2,6-di­chloro­phenyl ring and the nitro-substituted benzene ring of the title mol­ecule along the *a*-axis direction (Figs. 2 ▸ and 3 ▸). Furthermore, these mol­ecules are linked by inter­molecular N—H⋯O and C—H⋯Cl hydrogen bonds, forming pairs of hydrogen-bonded mol­ecular layers parallel to (20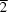) (Tables 1 ▸ and 2 ▸; Figs. 4 ▸ and 5 ▸). There is also a C—Cl⋯*Cg* inter­action [Cl1⋯*Cg*2(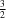 − *x*, 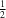 + *y*, 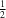 − *z*) = 3.9026 (14) Å; C2—Cl1⋯*Cg*2 = 64.12 (10)°]. As a result of the large Cl ⋯ *Cg*2 distance and acute C—Cl⋯*Cg*2 angle, this inter­action is only weak.

**Figure 6:**
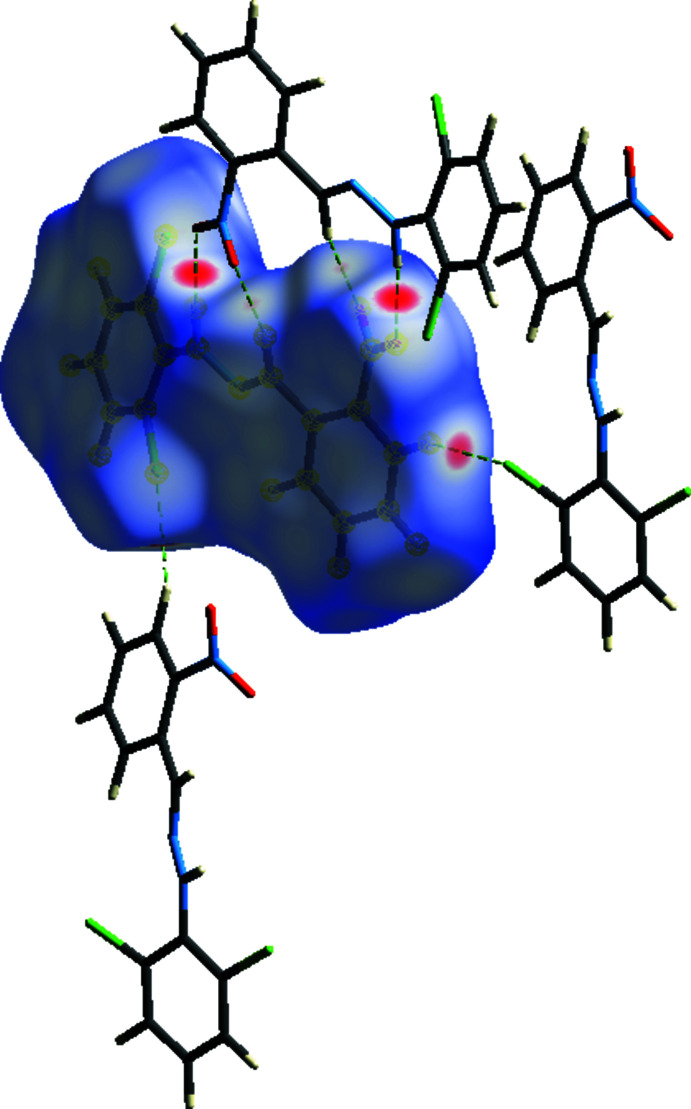
A view of the Hirshfeld surface mapped for the title compound over *d*_norm_ in the range −0.1980 to 1.3500 arbitrary units.

**Figure 7:**
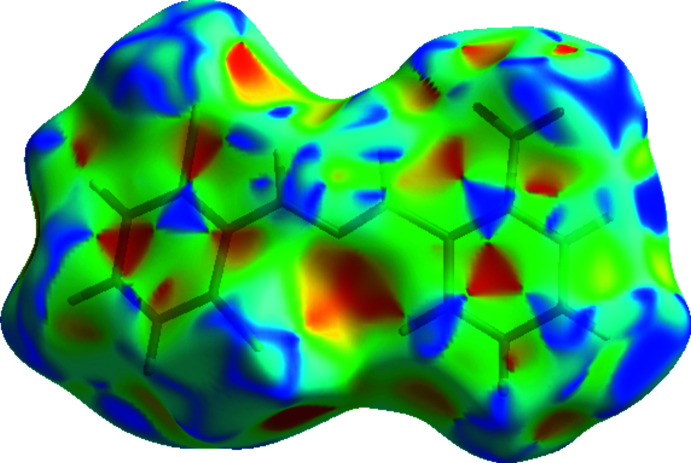
View of the three-dimensional Hirshfeld surface of the title compound plotted over shape-index.

Hirshfeld surface analysis was used to analyse the various inter­molecular inter­actions in the title compound, through mapping the normalized contact distance (*d*_norm_) using *CrystalExplorer* (Turner *et al.*, 2017 ▸; Spackman & Jayatilaka, 2009 ▸). The Hirshfeld surface mapped over *d*_norm_ using a standard surface resolution with a fixed colour scale of −0.1980 (red) to 1.3500 (blue) a.u. is shown in Fig. 6 ▸. The white surface indicates contacts with distances equal to the sum of van der Waals radii, and the red and blue colours indicate distances shorter (in close contact) or longer (distant contact) than the van der Waals radii, respectively (Venkatesan *et al.*, 2016 ▸). The dark-red spots on the *d*_norm_ surface arise as a result of short inter­atomic contacts (Table 2 ▸), while the other weaker inter­molecular inter­actions appear as light-red spots. The red points, which represent closer contacts and negative *d*_norm_ values on the surface, correspond to the C—H⋯O and C—H⋯Cl inter­actions. The shape-index of the Hirshfeld surface is a tool for visualizing the π–π stacking by the presence of adjacent red and blue triangles; if there are no such triangles, then there are no π–π inter­actions. The plot of the Hirshfeld surface mapped over shape-index shown in Fig. 7 ▸ clearly suggests that there are π–π inter­actions in the crystal packing of the title compound.

**Figure 8:**
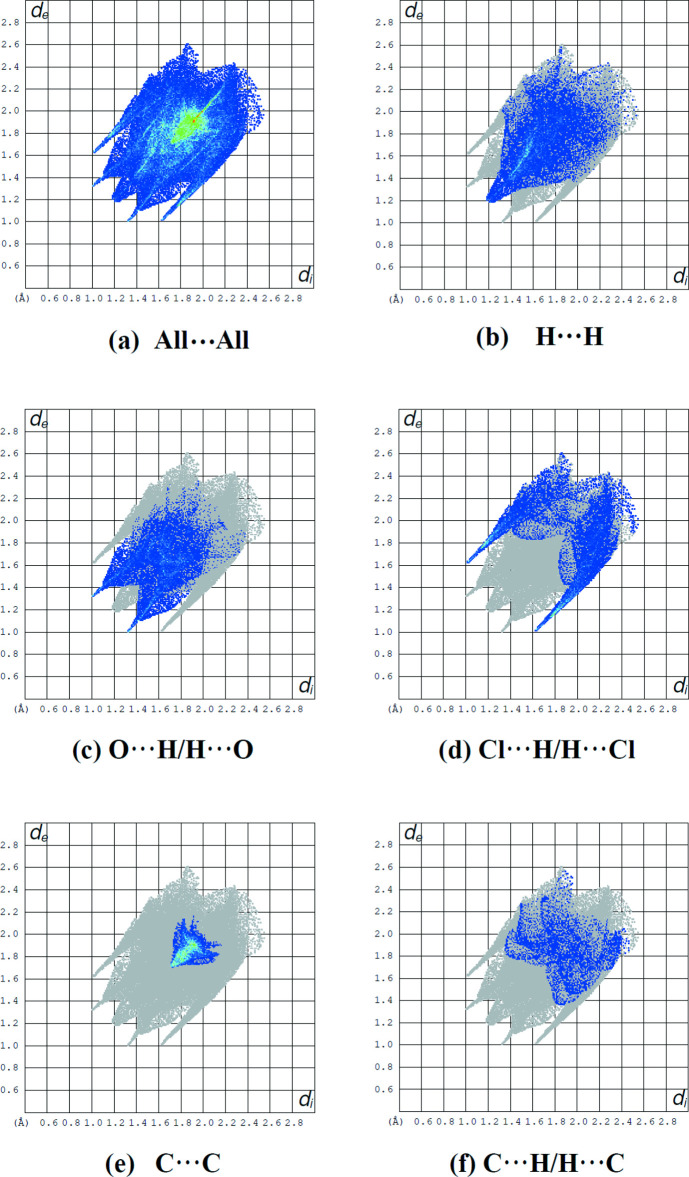
(*a*) The full two-dimensional fingerprint plot for the title compound and (*b*)–(*f*) those delineated into H⋯H, O⋯H/H⋯O, Cl⋯H/H⋯Cl, C⋯C and C⋯H/H⋯C contacts, respectively.

The percentage contributions of various contacts to the total Hirshfeld surface are listed in Table 3 ▸ and shown in the two-dimensional fingerprint plots in Fig. 8 ▸. As revealed by the two-dimensional fingerprint plots (Fig. 8 ▸), the crystal packing is dominated by H⋯H contacts, representing van der Waals inter­actions (23.0% contribution to the overall surface), followed by O⋯H and Cl⋯H inter­actions, which contribute 20.1% and 19.0%, respectively.

## Database survey   

Six compounds closely resemble the title compound, *viz*. 1-(2,4-di­nitro­phen­yl)-2-[(*E*)-(3,4,5-tri­meth­oxy­benzyl­idene)hydrazine] (CSD refcode GISJAV; Chantrapromma *et al.*, 2014 ▸), (*E*)-1-(2,4-di­nitro­phen­yl)-2-[1-(3-meth­oxy­phen­yl)eth­yl­idene]hydrazine (XEBCEO; Fun *et al.*, 2012 ▸), 1-(2,4-di­nitro­phen­yl)-2-[(*E*)-2,4,5-tri­meth­oxy­benzyl­idene]hydrazine (AFUSEB; Fun *et al.*, 2013 ▸), (*E*)-1-(2,4-di­nitro­phen­yl)-2-(1-(2-meth­oxy­phen­yl)ethyl­idene)hydrazine (OBUJAY; Fun *et al.*, 2011 ▸), (*E*)-1-(2,4-di­nitro­phen­yl)-2-[1-(3-fluoro­phen­yl)ethyl­idene]hydrazine (PAVKAA; Chantrapromma *et al.*, 2012 ▸) and (*E*)-1-(2,4-di­nitro­phen­yl)-2-[1-(2-nitro­phen­yl)ethyl­idene]hydrazine (YAHRUW; Nilwanna *et al.*, 2011 ▸). All bond lengths (Allen *et al.*, 1987 ▸) and angles for the title compound are within normal ranges and are comparable to those observed in these structures. In each one, the configuration of the imine C=N bond is *E*.

## Synthesis and crystallization   

The title compound was synthesized according to the reported method (Atioğlu *et al.*, 2019 ▸; Maharramov *et al.*, 2018 ▸; Shixaliyev *et al.*, 2018 ▸, 2019 ▸). A mixture of 2-nitro­benzaldehyde (10 mmol), CH_3_COONa (0.82 g), ethanol (50 mL) and (2,6-di­chloro­phen­yl)hydrazine (10.2 mmol) was refluxed at 353 K under stirring for 2 h. The reaction mixture was cooled to room temperature and water (50 mL) was added to give a precipitate of the crude product, which was filtered off, washed with diluted ethanol (1:1 with water) and dried *in vacuo* using a rotary evaporator. Crystals suitable for X-ray analysis were obtained by slow evaporation of an ethanol solution.

Title compound: orange solid (90%); m.p. 398 K. Analysis calculated for C_13_H_9_Cl_2_N_3_O_2_ (*M =* 310.13): C 50.35, H 2.93, N 13.55; found: C 50.27, H 2.86, N 13.54%. ^1^H NMR (300 MHz, DMSO-*d*_6_): *δ* 10.20 (1H, –NH), 8.41 (1H, –CH), 7.13–8.08 (7H, aromatic). ^13^C NMR (75 MHz, DMSO-*d*_6_): *δ* 147.47, 137.80, 133.76, 133.32, 130.17, 129.85, 129.16, 128.00, 127.08, 125.86, 124.96. ESI–MS: *m/z*: 311.08 [*M*+H]^+^.

## Refinement   

Crystal data, data collection and structure refinement details are summarized in Table 4 ▸. All H atoms were refined using a riding model with *d*(C—H) = 0.93 Å, *d*(N—H) = 0.95 Å and *U*_iso_ = 1.2*U*_eq_(N,C).

## Supplementary Material

Crystal structure: contains datablock(s) I. DOI: 10.1107/S2056989020008567/vm2235sup1.cif

Structure factors: contains datablock(s) I. DOI: 10.1107/S2056989020008567/vm2235Isup2.hkl

Supporting information file. DOI: 10.1107/S2056989020008567/vm2235Isup3.cml

CCDC reference: 2012294

Additional supporting information:  crystallographic information; 3D view; checkCIF report

## Figures and Tables

**Figure 3:**
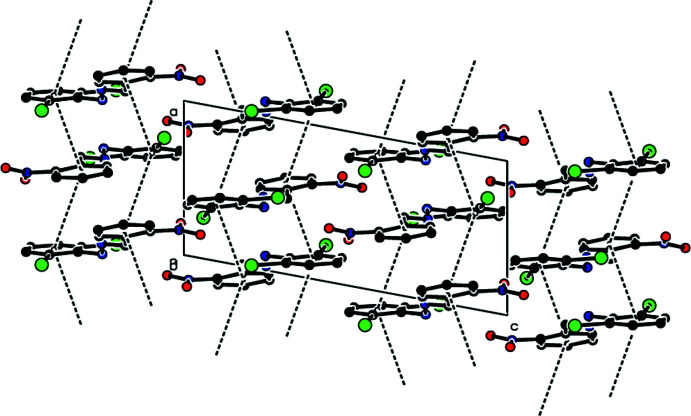
A partial view of π–π stacking inter­actions in the crystal packing of the title compound viewed along the *b* axis.

**Figure 5:**
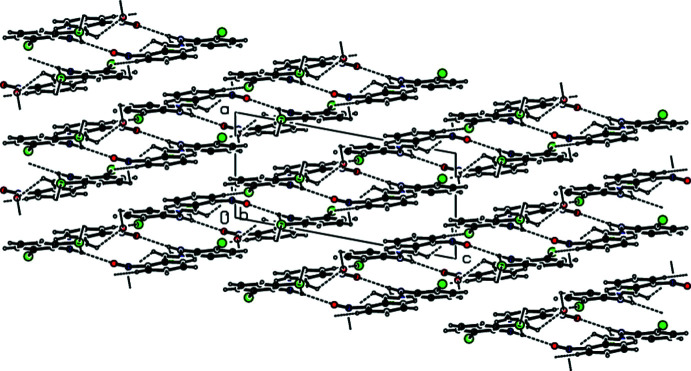
A general view of the crystal packing with the hydrogen bonds and contacts along the *b* axis of the title compound, forming pairs of hydrogen-bonded mol­ecular layers parallel to (20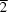).

**Table 1:** Hydrogen-bond geometry (Å, °) *Cg*2 is the centroid of the C8–C13 ring.

*D*—H⋯*A*	*D*—H	H⋯*A*	*D*⋯*A*	*D*—H⋯*A*
N1—H1*N*⋯Cl1	0.95	2.48	2.939 (2)	110
N1—H1*N*⋯O2^i^	0.95	2.40	3.327 (3)	166
C7—H7*A*⋯O1	0.93	2.34	2.774 (4)	108
C12—H12*A*⋯Cl2^ii^	0.93	2.80	3.679 (3)	157
C2—Cl1⋯*Cg*2^iii^	1.73 (1)	3.90 (1)	3.511 (3)	64 (1)

Symmetry codes: (i) 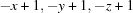; (ii) 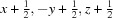; (iii) 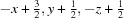.

**Table 2:** Summary of short inter­atomic contacts (Å) in the title compound

Contact	Distance	Symmetry operation
Cl1⋯H11*A*	3.06	*x*, 1 + *y*, *z*
C2⋯C8	3.464 (4)	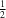 − *x*, 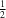 + *y*, 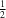 − *z*
H1*N*⋯O2	2.40	1 − *x*, 1 − *y*, 1 − *z*
O1⋯H4*A*	2.68	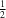 + *x*, 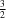 − *y*, 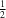 + *z*
Cl2⋯H12*A*	2.80	− 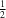 + *x*, 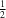 − *y*, − 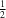 + *z*
N3⋯C4	3.447 (4)	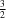 − *x*, − 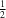 + *y*, 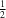 − *z*

**Table 3:** Percentage contributions of inter­atomic contacts to the Hirshfeld surface for the title compound

Contact	Percentage contribution
H⋯H	23.0
O⋯H/H⋯O	20.1
Cl⋯H/H⋯Cl	19.0
C⋯C	11.2
H⋯C/C⋯H	8.0
N⋯H/H⋯N	5.5
Cl⋯Cl	3.3
N⋯C/C⋯N	3.1
Cl⋯C/C⋯Cl	3.0
O⋯C/C⋯O	1.4
Cl⋯O/O⋯Cl	1.3
Cl⋯N/N⋯Cl	0.8
O⋯O	0.2
O⋯N/N⋯O	0.1

**Table 4:** Experimental details

Crystal data	
Chemical formula	C_13_H_9_Cl_2_N_3_O_2_
*M*_r_	310.13
Crystal system, space group	Monoclinic, *P*2_1_/*n*
Temperature (K)	296
*a*, *b*, *c* (Å)	7.1138 (4), 12.6827 (6), 15.1613 (8)
β (°)	100.571 (2)
*V* (Å^3^)	1344.67 (12)
*Z*	4
Radiation type	Mo *K*α
μ (mm^−1^)	0.49
Crystal size (mm)	0.26 × 0.22 × 0.18

Data collection	
Diffractometer	Bruker APEXII CCD
Absorption correction	Multi-scan (*SADABS*; Bruker, 2003 ▸)
*T*_min_, *T*_max_	0.868, 0.906
No. of measured, independent and observed [*I* > 2σ(*I*)] reflections	22007, 2521, 2184
*R*_int_	0.057
(sin θ/λ)_max_ (Å^−1^)	0.617

Refinement	
*R*[*F* ^2^ > 2σ(*F* ^2^)], *wR*(*F* ^2^), *S*	0.052, 0.118, 1.07
No. of reflections	2521
No. of parameters	181
H-atom treatment	H-atom parameters constrained
Δρ_max_, Δρ_min_ (e Å^−3^)	0.32, −0.40

Computer programs: *APEX3* and *SAINT* (Bruker, 2007 ▸), *SHELXT2016/6* (Sheldrick, 2015*a* ▸), *SHELXL2016/6* (Sheldrick, 2015*b* ▸), *ORTEP-3 for Windows* (Farrugia, 2012 ▸) and *PLATON* (Spek, 2020 ▸).

## supplementary crystallographic information

### Crystal data

**Table:**

C_13_H_9_Cl_2_N_3_O_2_	*F*(000) = 632
*M**_r_* = 310.13	*D*_x_ = 1.532 Mg m^−^^3^
Monoclinic, *P*2_1_/*n*	Mo *K*α radiation, λ = 0.71073 Å
*a* = 7.1138 (4) Å	Cell parameters from 9979 reflections
*b* = 12.6827 (6) Å	θ = 2.7–27.9°
*c* = 15.1613 (8) Å	µ = 0.49 mm^−^^1^
β = 100.571 (2)°	*T* = 296 K
*V* = 1344.67 (12) Å^3^	Plate, orange
*Z* = 4	0.26 × 0.22 × 0.18 mm

### Data collection

**Table:**

Bruker APEXII CCD diffractometer	2184 reflections with *I* > 2σ(*I*)
φ and ω scans	*R*_int_ = 0.057
Absorption correction: multi-scan (SADABS; Bruker, 2003)	θ_max_ = 26.0°, θ_min_ = 2.7°
*T*_min_ = 0.868, *T*_max_ = 0.906	*h* = −8→8
22007 measured reflections	*k* = −15→15
2521 independent reflections	*l* = −18→18

### Refinement

**Table:**

Refinement on *F*^2^	Primary atom site location: structure-invariant direct methods
Least-squares matrix: full	Secondary atom site location: difference Fourier map
*R*[*F*^2^ > 2σ(*F*^2^)] = 0.052	Hydrogen site location: mixed
*wR*(*F*^2^) = 0.118	H-atom parameters constrained
*S* = 1.07	*w* = 1/[σ^2^(*F*_o_^2^) + (0.026*P*)^2^ + 1.7039*P*] where *P* = (*F*_o_^2^ + 2*F*_c_^2^)/3
2521 reflections	(Δ/σ)_max_ < 0.001
181 parameters	Δρ_max_ = 0.32 e Å^−^^3^
0 restraints	Δρ_min_ = −0.40 e Å^−^^3^

### Special details

**Table:**

Geometry. All esds (except the esd in the dihedral angle between two l.s. planes) are estimated using the full covariance matrix. The cell esds are taken into account individually in the estimation of esds in distances, angles and torsion angles; correlations between esds in cell parameters are only used when they are defined by crystal symmetry. An approximate (isotropic) treatment of cell esds is used for estimating esds involving l.s. planes.

### Fractional atomic coordinates and isotropic or equivalent isotropic displacement parameters (Å^2^)

**Table:**

	*x*	*y*	*z*	*U*_iso_*/*U*_eq_	
Cl1	0.48623 (11)	0.84495 (6)	0.29065 (5)	0.0562 (2)	
Cl2	0.26473 (16)	0.52094 (7)	0.06054 (6)	0.0807 (3)	
O1	0.7013 (4)	0.44141 (18)	0.49042 (15)	0.0702 (7)	
O2	0.6516 (4)	0.2943 (2)	0.55110 (15)	0.0863 (8)	
N1	0.4068 (4)	0.62149 (17)	0.24789 (15)	0.0473 (6)	
H1N	0.396165	0.657432	0.301808	0.057*	
N2	0.4727 (3)	0.52096 (16)	0.24606 (15)	0.0419 (5)	
N3	0.6564 (3)	0.3486 (2)	0.48539 (15)	0.0509 (6)	
C1	0.3930 (4)	0.6850 (2)	0.17229 (17)	0.0405 (6)	
C2	0.4275 (4)	0.7938 (2)	0.18315 (18)	0.0410 (6)	
C3	0.4131 (4)	0.8620 (2)	0.1116 (2)	0.0530 (7)	
H3A	0.435083	0.933692	0.121363	0.064*	
C4	0.3661 (5)	0.8234 (3)	0.0258 (2)	0.0623 (9)	
H4A	0.359130	0.868645	−0.023006	0.075*	
C5	0.3296 (4)	0.7177 (3)	0.0122 (2)	0.0594 (8)	
H5A	0.298464	0.691722	−0.046044	0.071*	
C6	0.3384 (4)	0.6497 (2)	0.08380 (19)	0.0487 (7)	
C7	0.4904 (4)	0.4688 (2)	0.31903 (18)	0.0414 (6)	
H7A	0.467933	0.500896	0.371280	0.050*	
C8	0.5469 (4)	0.35755 (19)	0.31939 (17)	0.0377 (5)	
C9	0.5193 (4)	0.3022 (2)	0.23815 (19)	0.0468 (6)	
H9A	0.473272	0.337842	0.184977	0.056*	
C10	0.5589 (4)	0.1961 (2)	0.2353 (2)	0.0565 (8)	
H10A	0.541762	0.161493	0.180264	0.068*	
C11	0.6239 (4)	0.1405 (2)	0.3132 (2)	0.0586 (8)	
H11A	0.649659	0.068772	0.310822	0.070*	
C12	0.6501 (4)	0.1916 (2)	0.3942 (2)	0.0515 (7)	
H12A	0.691729	0.154697	0.447219	0.062*	
C13	0.6139 (4)	0.2987 (2)	0.39640 (17)	0.0396 (6)	

### Atomic displacement parameters (Å^2^)

**Table:**

	*U*^11^	*U*^22^	*U*^33^	*U*^12^	*U*^13^	*U*^23^
Cl1	0.0685 (5)	0.0450 (4)	0.0534 (4)	−0.0010 (3)	0.0064 (3)	−0.0065 (3)
Cl2	0.1061 (8)	0.0636 (5)	0.0648 (5)	−0.0150 (5)	−0.0046 (5)	−0.0194 (4)
O1	0.0956 (18)	0.0501 (13)	0.0590 (14)	−0.0025 (12)	−0.0014 (12)	−0.0147 (11)
O2	0.130 (2)	0.0877 (18)	0.0400 (12)	−0.0075 (17)	0.0109 (13)	0.0151 (12)
N1	0.0694 (16)	0.0337 (11)	0.0405 (12)	0.0096 (10)	0.0143 (11)	0.0029 (9)
N2	0.0482 (13)	0.0343 (11)	0.0444 (12)	0.0006 (9)	0.0112 (10)	0.0012 (9)
N3	0.0540 (14)	0.0554 (15)	0.0415 (13)	0.0043 (11)	0.0042 (11)	0.0005 (11)
C1	0.0391 (13)	0.0418 (14)	0.0398 (13)	0.0053 (11)	0.0053 (11)	0.0044 (11)
C2	0.0384 (13)	0.0408 (13)	0.0440 (14)	0.0039 (11)	0.0077 (11)	0.0041 (11)
C3	0.0544 (17)	0.0450 (16)	0.0610 (18)	0.0080 (13)	0.0140 (14)	0.0148 (14)
C4	0.064 (2)	0.071 (2)	0.0523 (18)	0.0132 (16)	0.0128 (15)	0.0224 (16)
C5	0.0589 (19)	0.079 (2)	0.0383 (15)	0.0090 (16)	0.0042 (13)	0.0023 (15)
C6	0.0492 (16)	0.0520 (16)	0.0427 (15)	0.0016 (13)	0.0023 (12)	−0.0015 (13)
C7	0.0481 (15)	0.0363 (13)	0.0414 (14)	0.0031 (11)	0.0120 (11)	−0.0008 (11)
C8	0.0381 (13)	0.0366 (13)	0.0398 (13)	−0.0018 (10)	0.0110 (10)	−0.0004 (10)
C9	0.0527 (16)	0.0465 (15)	0.0421 (14)	0.0006 (12)	0.0111 (12)	−0.0035 (12)
C10	0.0641 (19)	0.0477 (16)	0.0602 (19)	−0.0038 (14)	0.0178 (15)	−0.0190 (15)
C11	0.0602 (19)	0.0317 (14)	0.085 (2)	−0.0011 (13)	0.0175 (17)	−0.0048 (15)
C12	0.0545 (17)	0.0377 (14)	0.0615 (18)	0.0005 (12)	0.0089 (14)	0.0091 (13)
C13	0.0401 (13)	0.0374 (13)	0.0415 (14)	−0.0018 (10)	0.0078 (11)	−0.0008 (11)

### Geometric parameters (Å, º)

**Table:**

Cl1—C2	1.732 (3)	C4—H4A	0.9300
Cl2—C6	1.731 (3)	C5—C6	1.380 (4)
O1—N3	1.218 (3)	C5—H5A	0.9300
O2—N3	1.217 (3)	C7—C8	1.467 (3)
N1—N2	1.361 (3)	C7—H7A	0.9300
N1—C1	1.390 (3)	C8—C13	1.394 (4)
N1—H1N	0.9510	C8—C9	1.400 (4)
N2—C7	1.275 (3)	C9—C10	1.377 (4)
N3—C13	1.471 (3)	C9—H9A	0.9300
C1—C6	1.400 (4)	C10—C11	1.381 (5)
C1—C2	1.406 (4)	C10—H10A	0.9300
C2—C3	1.376 (4)	C11—C12	1.371 (4)
C3—C4	1.374 (4)	C11—H11A	0.9300
C3—H3A	0.9300	C12—C13	1.384 (4)
C4—C5	1.374 (5)	C12—H12A	0.9300
			
N2—N1—C1	119.9 (2)	C5—C6—Cl2	117.5 (2)
N2—N1—H1N	123.3	C1—C6—Cl2	121.2 (2)
C1—N1—H1N	115.2	N2—C7—C8	119.0 (2)
C7—N2—N1	116.6 (2)	N2—C7—H7A	120.5
O2—N3—O1	122.8 (3)	C8—C7—H7A	120.5
O2—N3—C13	118.4 (3)	C13—C8—C9	116.1 (2)
O1—N3—C13	118.7 (2)	C13—C8—C7	124.7 (2)
N1—C1—C6	124.7 (2)	C9—C8—C7	119.0 (2)
N1—C1—C2	119.2 (2)	C10—C9—C8	121.4 (3)
C6—C1—C2	116.0 (2)	C10—C9—H9A	119.3
C3—C2—C1	122.6 (3)	C8—C9—H9A	119.3
C3—C2—Cl1	118.5 (2)	C9—C10—C11	120.6 (3)
C1—C2—Cl1	119.0 (2)	C9—C10—H10A	119.7
C4—C3—C2	119.6 (3)	C11—C10—H10A	119.7
C4—C3—H3A	120.2	C12—C11—C10	119.7 (3)
C2—C3—H3A	120.2	C12—C11—H11A	120.2
C5—C4—C3	119.8 (3)	C10—C11—H11A	120.2
C5—C4—H4A	120.1	C11—C12—C13	119.3 (3)
C3—C4—H4A	120.1	C11—C12—H12A	120.3
C4—C5—C6	120.8 (3)	C13—C12—H12A	120.3
C4—C5—H5A	119.6	C12—C13—C8	122.8 (3)
C6—C5—H5A	119.6	C12—C13—N3	115.9 (3)
C5—C6—C1	121.2 (3)	C8—C13—N3	121.3 (2)
			
C1—N1—N2—C7	176.7 (2)	N2—C7—C8—C13	164.3 (3)
N2—N1—C1—C6	37.1 (4)	N2—C7—C8—C9	−20.8 (4)
N2—N1—C1—C2	−145.8 (2)	C13—C8—C9—C10	−0.8 (4)
N1—C1—C2—C3	−178.8 (2)	C7—C8—C9—C10	−176.0 (3)
C6—C1—C2—C3	−1.4 (4)	C8—C9—C10—C11	1.3 (5)
N1—C1—C2—Cl1	0.1 (3)	C9—C10—C11—C12	−0.3 (5)
C6—C1—C2—Cl1	177.4 (2)	C10—C11—C12—C13	−1.1 (5)
C1—C2—C3—C4	−1.0 (4)	C11—C12—C13—C8	1.6 (4)
Cl1—C2—C3—C4	−179.8 (2)	C11—C12—C13—N3	−176.6 (3)
C2—C3—C4—C5	1.5 (5)	C9—C8—C13—C12	−0.7 (4)
C3—C4—C5—C6	0.3 (5)	C7—C8—C13—C12	174.3 (3)
C4—C5—C6—C1	−2.9 (5)	C9—C8—C13—N3	177.4 (2)
C4—C5—C6—Cl2	173.1 (3)	C7—C8—C13—N3	−7.7 (4)
N1—C1—C6—C5	−179.5 (3)	O2—N3—C13—C12	−26.1 (4)
C2—C1—C6—C5	3.3 (4)	O1—N3—C13—C12	151.3 (3)
N1—C1—C6—Cl2	4.7 (4)	O2—N3—C13—C8	155.7 (3)
C2—C1—C6—Cl2	−172.5 (2)	O1—N3—C13—C8	−26.9 (4)
N1—N2—C7—C8	175.4 (2)		

### Hydrogen-bond geometry (Å, º)

*Cg*2 is the centroid of the C8–C13 ring.

**Table:**

*D*—H···*A*	*D*—H	H···*A*	*D*···*A*	*D*—H···*A*
N1—H1*N*···Cl1	0.95	2.48	2.939 (2)	110
N1—H1*N*···O2^i^	0.95	2.40	3.327 (3)	166
C7—H7*A*···O1	0.93	2.34	2.774 (4)	108
C12—H12*A*···Cl2^ii^	0.93	2.80	3.679 (3)	157
C2—Cl1···*Cg*2^iii^	1.73 (1)	3.90 (1)	3.511 (3)	64 (1)

Symmetry codes: (i) −*x*+1, −*y*+1, −*z*+1; (ii) *x*+1/2, −*y*+1/2, *z*+1/2; (iii) −*x*+3/2, *y*+1/2, −*z*+1/2.
